# Role of Ultrasound in the Identification of Longitudinal Axis in Soft-Tissue Foreign Body Extraction

**DOI:** 10.5811/westjem.2016.8.30988

**Published:** 2016-09-29

**Authors:** Al Lulla, Taryn Whitman, Ricky Amii, Alan T. Chiem

**Affiliations:** * Olive View-UCLA Medical Center, Department of Emergency Medicine, Sylmar, California; † California State University, Los Angeles, Department of Biological Sciences, Los Angeles, California

## Abstract

Identification and retrieval of soft-tissue foreign bodies (STFB) poses significant challenges in the emergency department. Prior studies have demonstrated the utility of ultrasound (US) in identification and retrieval of STFBs, including radiolucent objects such as wood. We present a case of STFB extraction that uses US to identify the longitudinal axis of the object. With the longitudinal axis identified, the foreign body can be excised by making an incision where the foreign body is closest to the skin. The importance of this technique as it pertains to minimizing surrounding tissue destruction and discomfort for patients has not been previously reported.

## INTRODUCTION

Soft-tissue foreign bodies (SFTB) are of important clinical significance in the emergency setting given the risk for significant inflammation, infection, impaired or prolonged wound healing and pain or discomfort for the patient.^1^ Physical exam, wound exploration and conventional radiography are ineffective means to identify or retrieve retained foreign bodies.^2^ In the emergency department (ED) setting, ultrasound (US) is a readily available tool that has been shown to be highly effective at identification of STFBs. In cadaveric and animal tissue studies, US has shown to have higher specificity and sensitivity than conventional imaging modalities such as plain film radiography in identification of STFBs.^3^

We present a case of a patient who was found to have a radiolucent wooden STFB that was detected and safely removed with the assistance of bedside US. Our retrieval technique emphasizes the importance of minimizing surrounding tissue destruction by using US to help identify the longitudinal axis of the foreign body.

## CASE REPORT

A 22-year-old woman with no significant past medical history presented to the ED with hip pain after hitting an old wooden table while walking by. The patient reported she felt something “go in” but was unable to retrieve the foreign body herself. She described the pain as dull, 3/10 in severity, non-radiating, and worse with ambulation. The patient’s vital signs on arrival were as follows: temperature 37.4°C; blood pressure 118/68 mmHg; heart rate 68 beats per minute; respiratory rate 14 breaths per minute; oxygen saturation 99% on room air. Physical exam of the hip revealed a 3mm puncture wound in the anterior-lateral thigh, approximately 10cm distal to the anterior-superior iliac spine. There was noted to be 1cm diameter of surrounding erythema; however, there was no fluctuance or induration that was appreciated. No foreign body was palpated on exam. No pain was elicited on passive extension of the hip or knee.

**Figure 1:**
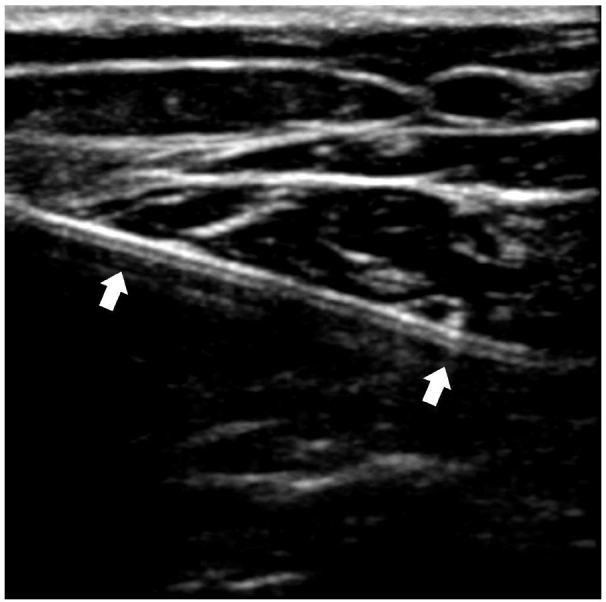
Ultrasound image of soft tissue foreign body (arrows) in longitudinal axis.

A plain film radiograph showed no evidence of retained foreign body. However, given clinical suspicion for a radiolucent retained foreign body, bedside US was done, which confirmed the presence of a foreign body in the anterior thigh (Figure 1). The US probe was placed in an orientation that allowed for direct visualization of the longitudinal axis of the foreign body. Using sterile technique at the bedside, a new tract was created using a scalpel and the foreign body was removed using forceps with traction applied along the longitudinal axis.

## DISCUSSION

Ultrasound has long been accepted to be a superior imaging modality in comparison to conventional radiography in the detection of radiolucent STFBs. In one retrospective study of 200 patients Anderson et al. showed that conventional radiographic studies identified wood foreign bodies only 15% of the time.^4^ Bray et al demonstrated that US has a sensitivity of 94% and specificity of 99% in the identification of STFBs.3 More recent studies, including a meta-analysis conducted by Davis et al showed that US has moderate sensitivity of 72% but still high specificity of 92% in the detection of STFBs.^5^ In addition, US can accurately measure the size of a lodged foreign body within ± 1 mm.^3^ Deeper foreign bodies can be retrieved with the aid of continuous US guidance. Bradley showed an 88% success rate in US-guided percutaneous removal of STFBs.^6^

**Figure 2:**
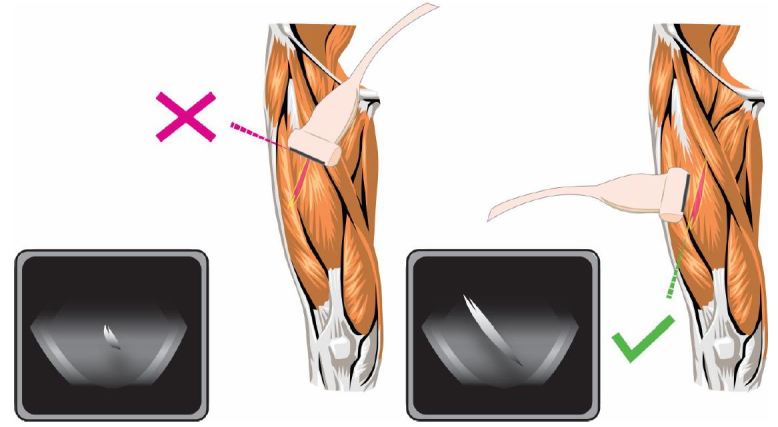
Approach to using ultrasound to aid with excision of soft-tissue foreign body. Left: Foreign body in transverse axis (incorrect approach). Right: Foreign body in longitudinal axis (correct approach).

The ability of ultrasound to accurately assess the length (longitudinal axis), width (transverse axis) and depth of the STFB offers significant advantage when it comes to removing the object.^7^ In particular, the ability of US to delineate the longitudinal axis of an object is critical because it decreases the amount of tissue dissection needed and reduces discomfort for the patient (Figure 2). As in the case of this particular patient, once the longitudinal axis is identified on US, an incision can be made at the site where the foreign body is closest to the skin.^8^ The foreign body is then grasped with forceps and traction is applied in parallel along the long axis of the foreign body. This allows for the object to easily slide along its long axis out of the soft tissue without causing unnecessary pain or tissue destruction.

It is possible to attempt to retrieve the foreign body by extending the incision from the entry wound.^9^ However, this poses certain risks and complications. On occasion, foreign bodies have been shown to migrate from the initial site of entry and can result in significant morbidity and mortality.^10^ In the event this occurs, it makes retrieval through the entry wound unfavorable due to the need for extensive dissection and further devitalization of surrounding soft-tissue structures. Instead, by locating the longitudinal axis of the STFB, the clinician is able to create a new tract that facilitates easy removal.
